# Surgical Trauma Comparison of Inframammary Fold versus Endoscopic Transaxillary Approaches in Breast Augmentation: A 7-Year Cohort Study

**DOI:** 10.1007/s00266-024-04619-5

**Published:** 2024-12-17

**Authors:** Zhaoyu Chen, Zhao Qiu, Jing Tong, Jie Yang, Chao Luo, Wenbin Jiang, Rongrong Wang, Jiaming Sun

**Affiliations:** 1 Department of Plastic Surgery, Union Hospital, Tongji Medical College, Huazhong University of Science and Technology, 1277 Jiefang Avenue, Wuhan, 430022 China; 2 Wuhan Clinical Research Center for Superficial Organ Reconstruction, Wuhan, 430022 China; 3 Department of Plastic Surgrey, Hefei BOE Hospital, Hefei, 230011 China

**Keywords:** Mammaplasty, Inframammary fold, Axillary, Surgical trauma

## Abstract

**Background:**

This retrospective cohort study aimed to assess differences in surgical trauma between the inframammary fold approach and endoscopic transaxillary approach in breast augmentation surgery.

**Methods:**

One hundred and ninety-four patients who underwent breast augmentation using either an inframammary fold or endoscopic transaxillary approach were enrolled. All procedures were primary and bilateral cases. Patients’ demographics and indicators, such as operation duration, postoperative volume of drainage, drainage duration, length of hospital stay, and postoperative pain scores, were observed and analyzed.

**Results:**

One hundred and five patients underwent inframammary fold incisions, while the remaining 89 received transaxillary incisions. The operation duration was significantly shorter in the inframammary fold group than in the transaxillary group, while the VAS scores were significantly lower (*p *< 0.001). Similarly, differences in the age and fertility status between the two groups were statistically significant (*p *< 0.05). However, no statistically significant differences were noted in the scores of the remaining indicators (*p *< 0.05).

**Conclusions:**

This research demonstrated that while patients in the endoscopic transaxillary group were typically younger, which is commonly hypothesized to result in superior results, the inframammary fold approach may offer a surgical option with reduced trauma and pain and concomitantly greater convenience and efficiency, yielding high satisfaction levels among Chinese women.

**Level of Evidence III:**

This journal requires that authors assign a level of evidence to each article. For a full description of these Evidence-Based Medicine ratings, please refer to the Table of Contents or the online Instructions to Authors www.springer.com/00266

## Introduction

Surgical trauma is an inevitable consequence of surgical procedures or interventions. Severe trauma not only induces pain in patients and influences operative effects but also increases the medication burden. Indeed, an increasing number of patients opt for lesser traumatic approaches regarding the selection of the type of aesthetic surgery. As is well documented, breast augmentation is the most common aesthetic surgical procedure, with about 280,692 procedures performed in 2019 and maintaining its leading position for several years [1–5].

Approaches for breast augmentation vary in techniques and incisions, including the transaxillary, peri-areolar, inframammary fold, and transumbilical approaches. Breast augmentation via the inframammary fold incision is the most widely adopted approach in Western countries owing to its simplicity and lower risk of contamination [6–8]. However, transaxillary and peri-areolar incisions are more frequently used in Asia, a preference attributed to the likelihood of Asian patients developing hypertrophic and dark-pigmented scars. Consequently, an incision hidden within the axilla or nearby areolar is favored. However, comparative studies investigating surgical trauma between endoscopic transaxillary and inframammary fold approaches are scarce.

This retrospective investigation aimed to compare surgical indicators between two approaches, including operation duration, postoperative volume of drainage, drainage duration, length of hospital stay, patient satisfaction level, and postoperative pain scores, to analyze and contrast the surgical trauma associated with breast augmentation performed by the same plastic surgeon.

## Patients and Methods

### Study Participants

The clinical data of consecutive patients attending our department between January 1, 2017, and December 31, 2023, with transaxillary and inframammary fold incisions were collected and examined. All recruited patients undergoing breast surgery for the first time opted for breast augmentation during this procedure. Patients were provided with various incision options during preoperative consultations and were instructed to select a specific incision type after being fully informed about the characteristics of each option. All patients in the study provided informed consent prior to their participation.

### Surgical Techniques

All surgical procedures were performed by Dr. Sun and his team. Patients were positioned in the upright position, with markings delineated along the midsternal line, parasternal line, midaxillary line, and inframammary fold line. Implants utilized in this study were exclusively textured silicone gel from Mentor and placed at the dual-plane level in both groups. In the transaxillary group, the surgical incision was precisely positioned along the skin crease line at the upper region of both armpits; an endoscope and endoscopic diathermy scissors were employed following dissection of the fascia of the pectoralis major muscle. The inframammary fold incision was meticulously aligned with the new breast fold contour. Prior to closing the operative area, negative pressure drainage tubes were inserted in all patients. Both groups underwent closure of incisions using interrupted sutures using 3–0 synthetic absorbable sutures within subcutaneous tissue and continuous suturing using 4–0 synthetic absorbable sutures within the mid-level dermis.

### Postoperative Evaluation

To assess surgical trauma, postoperative data were recorded, including operation duration (from surgery incision to dressing time), postoperative volume of drainage, drainage duration, hospital stay, and postoperative pain scores using the visual analogs scale (VAS). Patient satisfaction was assessed one year after surgery. Pictures of the patient’s breasts were captured before and after the procedure from various angles, including frontal, semi-profile, and bilateral views. The distance between the patient and the photographer remained constant to ensure accuracy and consistency.

### Statistical Analysis

Statistical analyses were conducted using SPSS version 27.0 by IBM Corp. in Armonk, NY. Descriptive statistics were presented as either the mean with standard deviation or as numbers. An independent t test was utilized to compare the mean of continuous variables such as patient age, implant volume, and operation duration. The Chi-square test was employed to evaluate differences between categorical variables such as fertility status. A two-tailed *p* value lower than 0.05 was considered statistically significant.

## Results

**Fig. 1:**
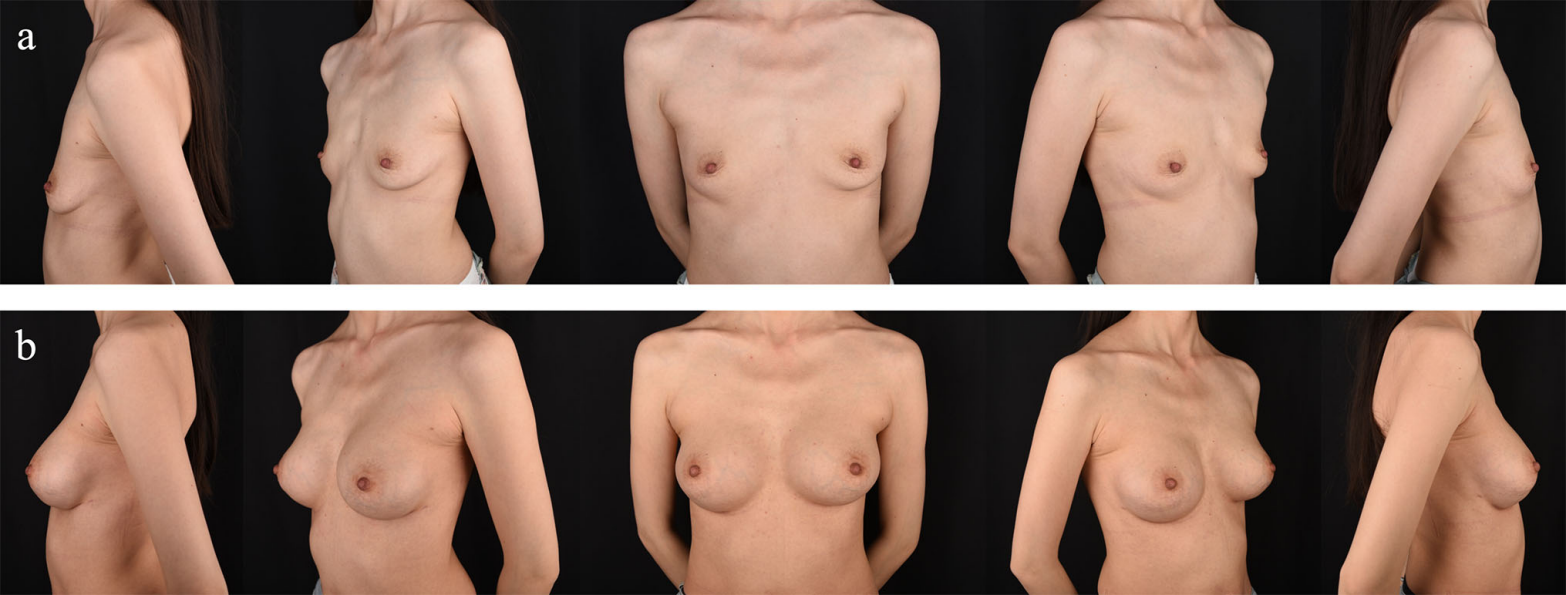
This 26-year-old parous patient with a BMI of 17.42 kg/m^2^ underwent inframammary fold incision. **a** Preoperative and **b** 3-month postoperative views

**Fig. 2:**
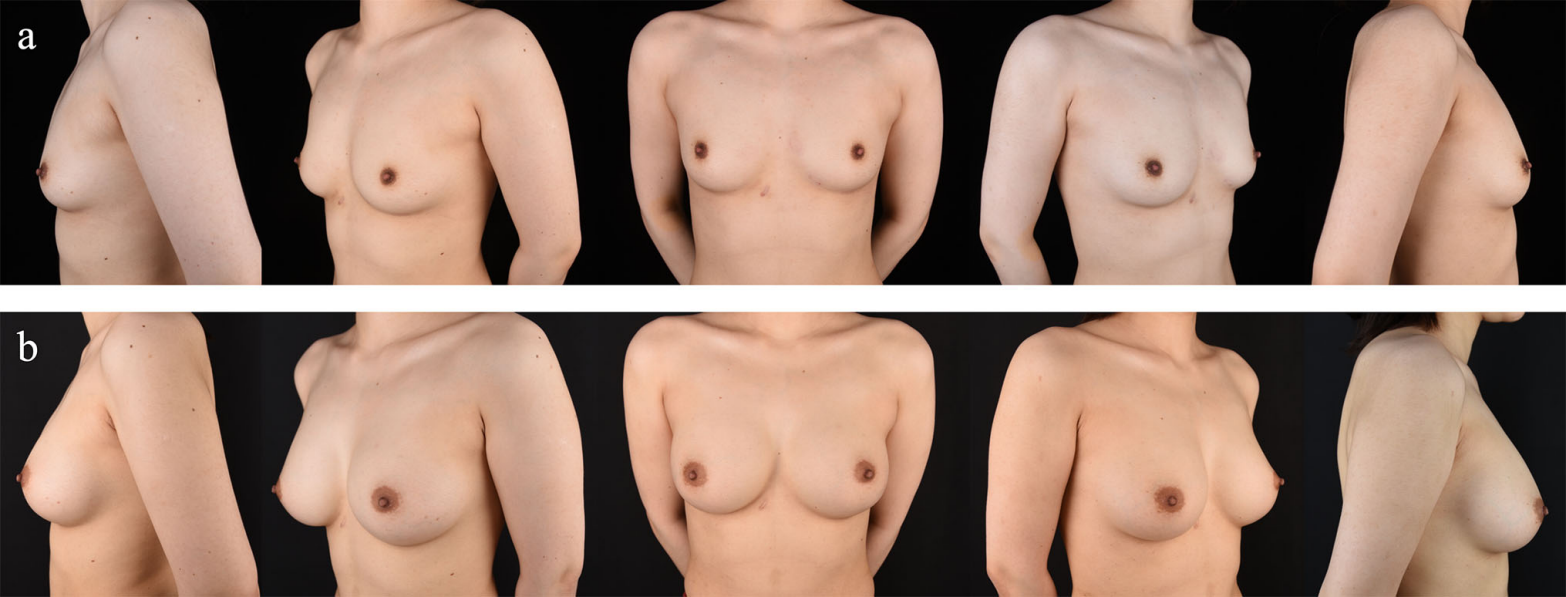
This 24-year-old parous patient with a BMI of 23.28 kg/m^2^ underwent transaxillary incision. **a** Preoperative and **b** 1-year postoperative views

One hundred and ninety-four patients of Chinese descent were included in this study, with an average age of 30.92 years, ranging from 18 to 61, and an average BMI of 19.44. One hundred and five patients underwent the surgical intervention with inframammary fold incisions, whereas 89 received transaxillary incisions. The details of patients are outlined in Table 1. The average age of the inframammary fold group was 32.70 years (range 21–61 years), while that of the transaxillary group was 28.83 years (range 18–45 years). This difference was statistically significant (*p *< 0.01). In the inframammary fold group, there were 79 parous and 26 nulliparous patients. In the transaxillary group, 53 patients were parous, and 36 were nulliparous. The difference in fertility status between the two groups was statistically different (*p *< 0.05). On the other hand, BMI and implant volume were comparable across the two groups. Likewise, baseline demographics and clinical features were similar between the two groups. Representative findings of each group are illustrated in Figs. 1 and 2.

**Table 1:** Patient baseline demographics

		Inframammary fold	Transaxillary	*p* value
Patients		105	89	
Age (year)		32.70±5.99	28.83±5.50	<0.01
BMI		19.20±1.293	19.64±2.49	0.132
Fertility status	Parity	79	53	<0.05
	Nulliparity	26	36	
Implant volume (cc)	Left	259.67±25.25	253.15±21.92	0.059
	Right	259.52±28.08	253.37±21.08	0.091

As listed in Table 2, operation duration, volume of drainage, drainage duration, total and postoperative hospital stay, visual analog scale (VAS), and satisfaction levels (including incision scars, the shape of the breasts, and their feel) were recorded to assess surgical trauma between the two groups. The operation duration was shorter in the inframammary fold group than in the transaxillary group, while the VAS scores were lower (both *p *< 0.001). In contrast, there were no statistically significant differences noted in the scores of the remaining indicators (*p *< 0.05).

**Table 2:** Surgical trauma and patient satisfaction of two incisions

	Inframammary fold	Transaxillary	*p* value
Patients	105	89	
Operation duration (min)	125.57±30.504	164.17±34.021	*p*<0.001
Drainage (mL)			
Day 1	182.71±137.409	202.53±93.161	0.25
Day 2	108.1±46.737	112.94±54.519	0.506
Day 3	79.52±39.744	71.03±39.643	0.139
Total	537.6±212.164	534.76±212.164	0.928
Unilateral	268.8±111.516	267.38±106.082	0.928
Drainage duration (day)	5.75±1.269	5.73±1.223	0.903
Total hospital stay (day)	7.41±1.697	7.75±1.674	0.159
Postoperative hospital stay (day)	6.06±1.413	6.25±1.282	0.331
VAS			
Day 1	1.73±1.012	2.72±1.243	*p*<0.001
Day 2	1.23±0.973	1.87±1.047	*p*<0.001
Day 3	0.89±0.923	1.54±1.149	*p*<0.001
Satisfaction			
Satisfied	91	79	0.676
Generally satisfied	9	5	
Unsatisfied	2	1	

Operation duration was (125.57 ± 30.504) min in the inframammary fold group and (164.17 ± 34.021) min in the transaxillary group. At the same time, the VAS score was 1.73 ± 1.012, 1.23 ± 0.973, 0.89 ± 0.923 in the inframammary fold group at days 1, 2, and 3 postoperatively, which was significantly lower than that in the transaxillary group, recorded as 2.72 ± 1.243, 1.87 ± 1.047, and 1.54 ± 1.149, respectively. Total drainage volume was (537.60 ± 223.031) mL and (534.76 ± 212.164) mL in inframammary fold and transaxillary groups, respectively. Meanwhile, drainage duration was (5.75 ± 1.269) days and (5.73 ± 1.223) days in the inframammary fold and transaxillary groups, respectively. Total length of hospital stay was (7.41 ± 1.697) days and (7.75 ± 1.697) days in the inframammary fold and transaxillary groups, respectively. In the inframammary fold group, 102 patients were successfully followed up and completed the questionnaire. Among them, 91 reported being satisfied, 9 were generally satisfied, and 2 were unsatisfied. In the transaxillary group, 85 patients were successfully followed up and completed the questionnaire. Among them, 79 patients were satisfied with the surgical results, five patients were generally satisfied, and one patient was not satisfied. No serious complications, such as infection, double bubble, implant displacement, rippling, rupture, and capsular contracture (defined as Baker scale grades III and IV), were observed throughout the study.

## Discussion

In the field of plastic surgery, the relationship between surgical trauma and surgical outcomes holds significant implications and can influence various aspects of recovery, patient satisfaction, and overall success. Breast augmentation is not only the most popular aesthetic surgery but also the most prevalent surgical procedure in the 18–64 age group [1]. A range of studies have emphasized the importance of minimizing surgical trauma in breast augmentation surgery. Tebbetts [9] and Adams [10] both stressed the significance of patient selection and tissue evaluation and refined surgical techniques to mitigate the risk of complications and reoperations. Schneider [11] and Wilson [12] highlight the role of pain management and infection prevention, respectively, in enhancing patient outcomes. These findings collectively underscore the need for a comprehensive approach to breast augmentation that prioritizes meticulous planning and the use of advanced surgical and postoperative techniques.

The most popular incision for breast augmentation in Western countries is the inframammary fold approach, which is favored for its minimal scarring and consistent results [13]. In Asian countries, the transaxillary approach is the predominant choice owing to its easily hidden scar and lower capsular contracture rate [14, 15]. Herein, surgical indicators were compared between two breast augmentation approaches, encompassing operation duration, hospital stay, postoperative drainage, pain, and patient satisfaction.

The duration of surgeries using the inframammary fold approach was significantly shorter than that employing the transaxillary approach. Surgical duration has been consistently linked to an increased risk of complications in plastic surgery, including medical and surgical complications [16, 17]. Besides, longer surgical durations are associated with a higher risk of morbidities, with a significant increase in the frequency of complications observed 3 h post-surgery [18]. Herein, no significant differences were observed in the rates of complications and length of hospitalization, potentially ascribed to the short duration of both surgeries. Of note, Lee [15] pointed out that the incidence of complications for both two incisions is similar.

Research has consistently demonstrated a significant relationship between surgical trauma and postoperative pain in the field of plastic surgery. Boekel [19] determined that higher postoperative pain scores were associated with a higher risk of complications, highlighting the need for personalized analgesia. Schoenbrunner [20] further supported this conclusion, emphasizing the importance of multimodal analgesia in managing postoperative pain and suggesting that a combination of medications and techniques can effectively minimize pain and optimize recovery. Surgeons play a crucial role in managing postoperative pain by implementing enhanced recovery after surgery (ERAS) protocols that involve a multimodal analgesia regimen that includes various non-opioid analgesic techniques such as local anesthetics, NSAIDs, COX-2 inhibitors, and other medications to reduce opioid consumption and improve patient outcomes [20]. Gramke [21] reported that 26% of patients experienced moderate to severe pain on the day of the operation, with its prevalence decreasing over the following days. In this study, NSAIDs were administered for pain management, and VAS was applied to assess postoperative pain intensity. Noteworthily, the scores of patients in the inframammary fold group were lower than those in the transaxillary group in the first three days, indicating that postoperative pain may be milder with an inframammary fold incision compared to a transaxillary incision. This may be related to the shorter surgical path, smaller dissection range, and ultimately lesser trauma elicited via the inframammary fold incision surgery.

Several factors have been identified as potential influencers of postoperative drainage volume in breast augmentation surgeries, including the surgical approach, instruments used, level of dissection, method of dressing, and negative pressure level of drainage devices [22–24]. In the two approaches included in the study, efforts were made to adjust for other confounders, using the same high-frequency electric knife equipment, negative pressure drainage devices, and dressing methods. Considering that the drainage tubes of some patients were removed on the fourth day after surgery, only the drainage volume from the first three days after surgery was statistically analyzed. In this study, no significant differences were found in the average daily drainage volume, total drainage volume, and duration of drainage tube retention after surgery between the groups undergoing dual-plane breast augmentation surgery through the transaxillary and inframammary fold approaches (*p *> 0.05). We postulate that these results may be attributed to the fact that although the inframammary fold approach had a shorter surgical path and smaller dissection range compared to the transaxillary approach, the cavity of patients where the implant was placed was separated from the dissected transaxillary cavity into upper and lower parts during postoperative compression dressing in the transaxillary group, with the drainage tube primarily placed in the lower region of the implant. Therefore, after postoperative compression dressing, the actual drainage area of the two groups may be consistent.

In this study, the average age of patients undergoing the endoscopic transaxillary approach was 28.83 years, which was lower than the average age of 32.70 years (*p *< 0.01) among those undergoing the endoscopic transaxillary approach. Among the sixty-two nulliparous patients, 36 patients (58.1%) opted for the endoscopic transaxillary approach, while the remaining 26 patients (41.9%) underwent the transaxillary approach. The rate of nulliparity was significantly higher in the transaxillary group compared with the inframammary fold group (*p* < 0.05). Notably, young, nulliparous patients were more inclined to opt for easily hidden endoscopic transaxillary incision for breast augmentation surgery, which is in line with the research findings of Nguyen [25]. Patients in both groups were highly satisfied with the surgical outcomes. Conversely, patients who were generally satisfied or unsatisfied were largely due to scarring. Sun [26–28] described that the axillary approach was the preferred option for more than half of Chinese women. Nonetheless, in their subsequent study, they validated that scars and patient satisfaction was comparable between the two approaches in the long-term following surgery. In addition, despite the older age of patients in the inframammary fold group, there was no increase in the risk of adverse surgical outcomes.

In summary, this study contributes to the growing body of evidence on the impact of surgical approach choice on breast augmentation outcomes. While the endoscopic transaxillary approach is favored by younger patients for its scar concealment, our findings suggest that the inframammary fold approach may offer significant advantages in reducing surgical trauma, postoperative pain, and recovery time. This approach appears to provide a practical and efficient option, particularly for patients prioritizing recovery ease over aesthetic considerations. Future research could further validate these findings by assessing long-term outcomes and exploring the impact of patient demographics on surgical approach preferences, ultimately guiding more personalized treatment strategies in breast augmentation.

## Conclusion

This retrospective study analyzed the clinical data of two groups of patients undergoing two different surgical approaches, comprehensively comparing operation duration, drainage volume, drainage duration, pain levels, and patient satisfaction. Our findings demonstrated that while patients in the endoscopic transaxillary group were generally younger, often assumed to lead to better outcomes, the inframammary fold approach may be less traumatic and pain, more convenient and efficient, and equally capable of achieving high satisfaction levels in Chinese women.
